# Information-Based Boundary Equilibrium Generative Adversarial Networks with Interpretable Representation Learning

**DOI:** 10.1155/2018/6465949

**Published:** 2018-10-17

**Authors:** Junghoon Hah, Woojin Lee, Jaewook Lee, Saerom Park

**Affiliations:** Industrial Engineering, Seoul National University, 1 Gwanakro, Gwanak-gu, Seoul 08826, Republic of Korea

## Abstract

This paper describes a new image generation algorithm based on generative adversarial network. With an information-theoretic extension to the autoencoder-based discriminator, this new algorithm is able to learn interpretable representations from the input images. Our model not only adversarially minimizes the Wasserstein distance-based losses of the discriminator and generator but also maximizes the mutual information between small subset of the latent variables and the observation. We also train our model with proportional control theory to keep the equilibrium between the discriminator and the generator balanced, and as a result, our generative adversarial network can mitigate the convergence problem. Through the experiments on real images, we validate our proposed method, which can manipulate the generated images as desired by controlling the latent codes of input variables. In addition, the visual qualities of produced images are effectively maintained, and the model can stably converge to the equilibrium. However, our model has a difficulty in learning disentangling factors because our model does not regularize the independence between the interpretable factors. Therefore, in the future, we will develop a generative model that can learn disentangling factors.

## 1. Introduction

The generative adversarial network (GAN) model that is one of the prominent generative models has been successfully applied to image generation task [1]. The basic idea of GAN is to adversarially train two different models, a generator and a discriminator. The generator aims to generate fake samples similar to real samples from random noise variables. Meanwhile, the discriminator learns to distinguish real samples from fake samples obtained by the generator. When a GAN model reaches the convergence, the generator can give indistinguishable samples with real samples. In addition, distributed representations of the inputs can be obtained from the hidden representations of the GAN model, which reflect the underlying factors of variation that generate the data [2]. However, the original GAN model has difficulties in training and modal collapse [3].

To overcome these problems, many improved models have been proposed [4–6]. While the original GAN model has a binary classification discriminator that distinguishes fake samples from real ones, the recent models construct more informative discriminator costs such as an autoencoder cost that assign lower cost to real samples and higher cost to fake ones [3, 6, 7]. Boundary Equilibrium GAN (BEGAN) [3] used an autoencoder cost as a discriminator cost and balanced adversarial network using proportional control theory, and it, as a result, converged to diverse images of the highest visual quality without a complex alternating training procedure. However, the learned representations of images are entangled and barely interpretable because each dimension in distributed representations does not have a specific meaning.

Unsupervised learning is a general problem, which requires extraction of some valuable information from unlabeled data. Representation learning is one popular framework, which tries to learn a representation from unlabeled data that can be easily decoded [8–10]. In generative models, if we can learn interpretable representations with methods of unsupervised learning, it can be useful for generating new data. In fact, there are many generative models that construct new data with high quality with arbitrarily bad representations [11]. However, good generative models, including the idea of unsupervised learning, are expected to learn an interpretable representation and synthesize new data that can be manipulated as desired.

On the assumption that a good representation can find the underlying causes from the samples and is interpretable, supervised or semisupervised generative models have been developed [12–17]. Early methods of representation learning were based on autoencoders or RBMs (Restricted Boltzmann Machines) [18, 19]. Recently, VAEs (Variational Autoencoders) [12] achieved splendid semisupervised results on MNIST dataset [20], and GANs learned image representation that enables linear algebra on coded space [4]. There were several attempts to learn disentangled or interpretable representations with supervised datasets [21]. Such methods train the model to match one class of representations and supplied label. Similar to that, adversarial autoencoders [22] and VAEs learned representations with class label separated from other variations. To avoid those methods that explicitly label the variations, weakly supervised methods were introduced.

Because VAE and GAN frameworks have been popularly used in generative modeling, many models for learning disentangled or interpretable representations based on them have been proposed [13, 16, 17]. In generative models, it is important that some factors can be manipulated to generate a new image. However, in supervised models, what disentangling factors are learned is determined when constructing a model. Therefore, the factors that can be manipulated are also determined. Mathieu et al. developed a conditional generative model for learning to disentangle the factors of variation [13]. Their model consisted of two kinds of factors of variations such as the specified factors and the remaining factors. The specified part is modeled as VAE framework, and the remaining part is modeled as GAN framework. Xiao et al. proposed DNA-GAN, a supervised learning model for learning disentangling factors of variation [17]. They iteratively trained the model to address the problem of unbalanced multiattribute datasets. Their model also consisted of attribute-relevant and attribute-irrelevant parts. Higgins et al. developed a Symbol-Concept Association Network (SCAN) which learns higher-level concepts based on disentangled visual primitives, and these concepts can be used to generate new images [23]. They measured the accuracy and diversity where high accuracy means that a model understands the meaning of symbol and high diversity means the samples have the variety in terms of the unspecified attributes. The SCAN model showed good performance on accuracy and diversity. Even if these models were effective in learning disentangled representations, they required the paired training data with labels and could not overcome the instability problem of GAN objective.

Meanwhile, there exists a method that learns disentangled representations with unsupervised learning. Unsupervised learning is more desirable because not only the labeling cost is high but also the labels annotated by human may be inconsistent and insufficient [24] Kim and Mnih proposed an unsupervised factor VAE, which enhanced disentanglement over *β*-VAE by introducing the total correlation penalty [24]. However, they conducted the experiment only for the artificial dataset. The hossRBM successfully learned disentangled representation in an unsupervised way on Toronto Face Dataset, which showed emotional changes in generated images. However, hossRBM can only learn discrete latent factors, and the computation cost grows exponentially. InfoGAN model made it possible to learn interpretable representation in purely unsupervised way by introducing the mutual information concept of latent code and input representation [25]. InfoGAN can learn both discrete and continuous latent factors as interpretable representations. Also InfoGAN usually requires the same amount of training time of typical GANs. Although this model learned interpretable and meaningful representations, it still had convergence or modal collapse problem because of binary classification discriminator. Therefore, we aim at developing a generative model that learns interpretable distributed representations and improves training GAN model.

In this study, we proposed IBEGAN model which introduces mutual information regularization between latent codes of the generator and latent representations of the autoencoder model of the discriminator. In our model, the training procedure follows the one of the BEGAN model for stable training and high visual quality.

In the remainder of the paper, we first review the related GAN models such as BEGAN and InfoGAN in Section 2. In Section 3, we describe our proposed model, information-based boundary equilibrium generative adversarial networks (IBEGANs), in terms of GAN objective functions and model architecture. In section 4, we evaluate whether our model can learn interpretable representations by manipulating the generated images through the latent codes in real-world image datasets, and the analyses of the image quality and the model convergence are performed. Finally, section 5 concludes this study.

## 2. Related Work

There are many GAN models to improve the generated input quality and obtain effective latent representations. Energy-Based GANs (EBGANs) [6] attempted to use an energy function to model the discriminator *D*(**x**) based on the autoencoder model. Deep Convolutional GANs (DCGANs) [4] first used convolutional layer architecture and produced significantly improved visual samples. Wasserstein GANs (WGANs) [26] proposed to use Wasserstein distance as a measure of distance, which led to stable convergence. In this section, we introduce two GAN models that improve training procedure and learn interpretable representation: BEGAN and InfoGAN.

### 2.1. Boundary Equilibrium Generative Adversarial Networks

BEGAN construct GAN objective by using Wasserstein distance for autoencoder model. This method balances the generator and discriminator and also provides a new approximate convergence measure. BEGAN has a simpler architecture and easier training procedure compared to other typical GANs. As a result, BEGAN can produce the samples of human faces, with the best visual quality.

BEGAN has proposed to match the loss distributions of autoencoders instead of matching data distributions directly. BEGAN uses the loss for a pixel wise autoencoder ℒ:  *ℝ*^*N*_*x*_^⟶*ℝ*^*N*_*x*_^ as:

(1) $$\begin{array}{c} ℒ\left(x\right)={\left|x-D\left(x\right)\right|}^{\eta }\;\text{where}\left\{\begin{array}{c} D\text{:}\;\;{\mathbb{R}}^{N_{x}}⟶{\mathbb{R}}^{N_{x}}\;\text{is}\;\;\mathrm{the}\;\;\mathrm{auto}-\text{encoder}\;\;\mathrm{function},\\ x\in {\mathbb{R}}^{N_{x}}\;\text{is}\;\;a\;\;\mathrm{sample}\;\;\mathrm{of}\;\;\mathrm{dimension}\;\;N^{x},\\ \eta \in \left\{1,2\right\}\;\mathrm{is}\;\;\mathrm{the}\;\;\mathrm{target}\;\;\mathrm{norm}. \end{array}\right. \end{array}$$

Let *ν*_1_, *ν*_2_ be loss distributions of autoencoders, *C*(*ν*_1_, *ν*_2_) be the set of couplings of *ν*_1_ and *ν*_2_, and *m*_1_, *m*_2_ ∈ *ℝ* be the respective means. Then, Wasserstein distance is bounded by Jensen's inequality as follows in [3]:

(2) $$\begin{array}{c} W\left(\nu_{1},\nu_{2}\right)=\underset{\gamma \in C\left(\nu_{1},\nu_{2}\right)}{\inf}E_{\left(\mu_{1},\mu_{2}\right)∼\gamma }\left[\left|\mu_{1}-\mu_{2}\right|\right]\geq \left|m_{1}-m_{2}\right|, \end{array}$$

where *μ*_1_, *μ*_2_ have the same marginal distributions with *ν*_1_, *ν*_2_.

From this inequality, GAN loss is designed to maximize the lower bound of Wasserstein distance between autoencoder losses. Let *θ*_D_ and *θ*_G_ be the parameters of the discriminator and the generator, respectively, ℒ_D_ and ℒ_G_ be the losses of the discriminator and the generator, respectively, and **z** be the latent representation from autoencoder model. Then, the GAN objective can be expressed as follows:

(3) $$\begin{array}{c} \left\{\begin{array}{cc} {ℒ}_{\text{D}}=ℒ\left(x\right)-ℒ\left(G\left(z_{\text{D}};\theta_{\text{G}}\right)\right), & \text{for}\;\;\theta_{\text{D}},\\ {ℒ}_{\text{G}}=-{ℒ}_{\text{D}}, & \text{for}\;\;\theta_{\text{G}}. \end{array}\right. \end{array}$$

In the GAN model, balancing losses of the discriminator and the generator is difficult because the discriminator usually wins over the generator easily. Therefore, BEGAN introduces a new hyperparameter *γ* ∈ [0,1] for stable convergence as well as balancing goal as follows:

(4) $$\begin{array}{c} \gamma =\frac{E\left[ℒ\left(G\left(z\right)\right)\right]}{E\left[ℒ\left(x\right)\right]}. \end{array}$$

If *γ* becomes lower, the discriminator will focus on learning how to autoencode the real images. So image diversity will decrease. So BEGAN called *γ* as the diversity ratio.

BEGAN proposed to use proportional control theory to keep the equilibrium balanced at *γ𝔼*[ℒ(**x**)]=*𝔼*[ℒ(*G*(**z**))] by using a new variable *k*_*i*_ ∈ [0,1] which controls how much to focus on the loss of the generator during gradient descent. Finally, the full BEGAN objective is as follows:

(5) $$\begin{array}{c} \left\{\begin{array}{cc} {ℒ}_{\text{D}}=ℒ\left(x\right)-k_{i}ℒ\left(G\left(z_{\text{D}}\right)\right), & \text{for}\;\;\theta_{\text{D}},\\ {ℒ}_{\text{G}}=ℒ\left(G\left(z_{\text{G}}\right)\right), & \text{for}\;\;\theta_{\text{G}},\\ k_{i+1}=k_{i}+\lambda_{\text{k}}\left(\gamma ℒ\left(x\right)-ℒ\left(G\left(z_{\text{G}}\right)\right)\right), & \text{for}\;\;\mathrm{each}\;\;\mathrm{training}\;\;\mathrm{step}\;\;i, \end{array}\right. \end{array}$$

where *λ*_*k*_ is the learning rate for *k*.

BEGAN has stable convergence and simple training procedure because it is not required to pre-train the discriminator and to train the discriminator and the generator alternatively. Nevertheless, this model cannot obtain interpretable representation which is useful to generate new samples from the generative model. Therefore, in the following section, we explain the InfoGAN model which learns the meaningful latent code.

### 2.2. Information Maximizing Generative Adversarial Networks

InfoGAN introduced the information theoretical modification to original GAN. It enabled the model to learn interpretable representations, which inspired our proposed method. InfoGAN relates the latent variable to the input variable by maximizing the lower bound of mutual information.

InfoGAN decomposes the input noise vector to the incompressible noise **z** and structured semantic latent code **c**. The latent code **c** is a concatenation of latent variables **c**_1_, **c**_2_,…, **c**_*N*_ that were assumed to have factored distribution *P*(**c**_1_, **c**_2_,…, **c**_*N*_)=∏_*i*=1_^*N*^*P*(**c**_*i*_). In InfoGAN model, the incompressible noise **z** and the latent code **c** are both fed to the generator network, so the form of the generator becomes *G*(**z**, **c**), but the generator can ignore the information that **c** contains. To solve this problem, the mutual information of **c** and *G*(**z**, **c**) is maximized to restrict the generator on using the noise in a highly entangled way, where the lower bound of mutual information *I*(**c**; *G*(**z**, **c**)) can be derived by defining an auxiliary distribution *Q*(**c**|**x**).

Hence the minimax game of InfoGAN is defined as follows:

(6) $$\begin{array}{c} \underset{\mathrm{G,Q}}{\min}\underset{D}{\max}{ℒ}_{\text{InfoGAN}}\left(G,D,Q\right)=ℒ\left(G,D\right)-\lambda L_{\text{I}}\left(G,Q\right), \end{array}$$

where *L*_*I*_(*G*, *Q*)=*H*(**c**)+*𝔼*_c∼*P*(**c**),**x**∼*G*(**z**, **c**)_[log *Q*(**c**|**x**)].

InfoGAN successfully captured disentangled representations in MNIST dataset, 3D rendered image dataset, and the SVHN dataset. Those tasks were all unsupervised tasks, and the interpretable representations learnt by InfoGAN were competitive with several representations learnt by existing supervised methods. However, InGAN could not be applied to high-quality image examples because the binary classification discriminator was used instead of pixelwise error. Therefore, we propose a new information-based GAN model effectively reflecting pixelwise error.

## 3. Proposed Method

In this study, we proposed the IBEGAN model to generate and control new images with high visual quality and diversity. We used an autoencoder model with pixelwise error and proportional control theory to construct the discriminator loss as BEGAN model to alleviate convergence and diversity-quality balance problems. However, in the autoencoder model, we cannot restrict the kinds of latent representation such as discrete or continuous variable. IBEGAN consists of both continuous latent variable and discrete latent code. Therefore, we construct a new model architecture to learn interpretable representations with the discriminator using the autoencoder model.

**Figure 1:**
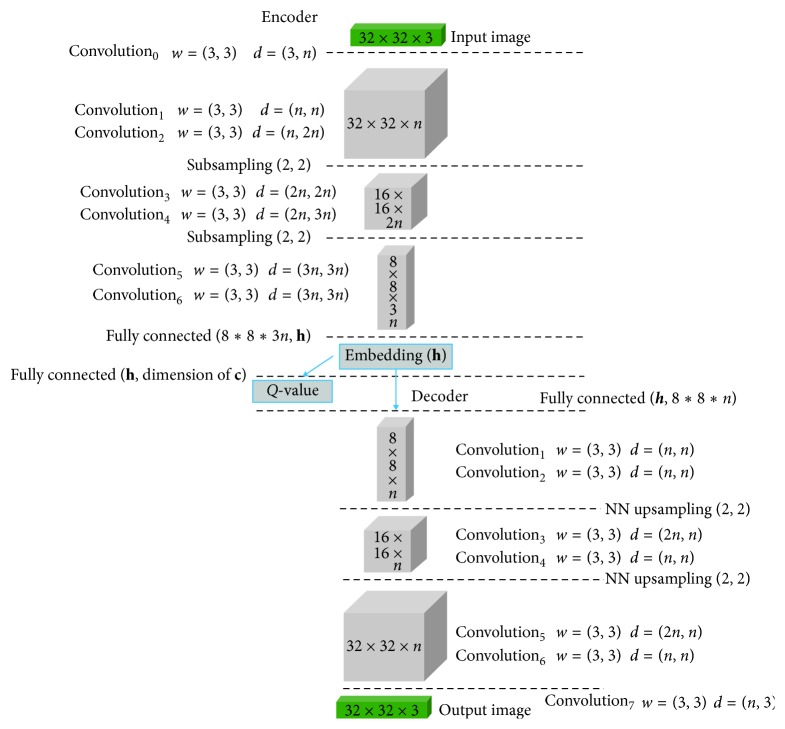
Network architecture of the discriminator.

**Figure 2:**
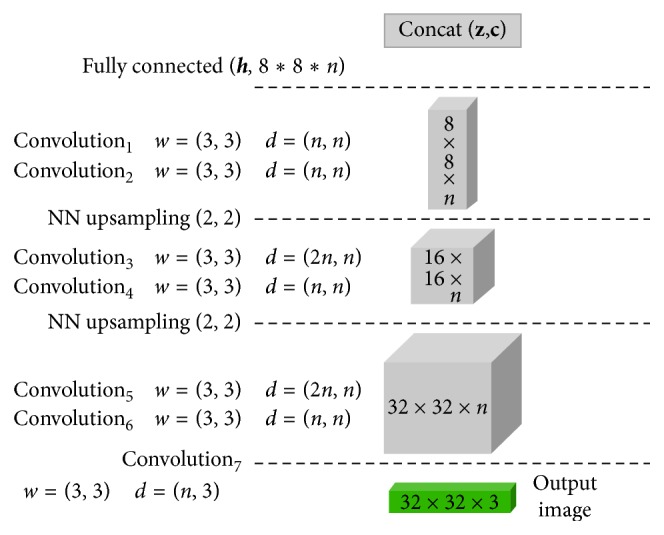
Network architecture of the generator.

First, our generator has two latent representations: incompressible noise **z** and latent code **c**, which are used for the inputs of the generator *G*(**z**, **c**), where **z** learns compact representation of image and **c** learns an interpretable factor that can manipulate generating images. An autoencoder model with pixelwise loss is constructed, where autoencoder loss distributions is used for constructing the losses of the discriminator and generator. Unlike the InfoGAN model, IBEGAN can obtain a reproducible encoding **h** because an image can be obtained by decoding **h** in our model. However, like the InfoGAN model, the posterior distribution should be approximated by *Q*(**c**|**x**) because we cannot explicitly estimate a posterior *P*(**c**|**x**). If a new network architecture is used for *Q*(**c**|**x**), computational cost will be increased, so we share the encoder network to construct the network of *Q*(**c**|**x**) where **q** can be obtained from **h**. In addition, we used a convolutional architecture to improve image quality. Figures 1 and 2 show the architecture of IBEGAN. The discriminator *D*:  ℛ^*N*_*x*_^⟶ℛ^*N*_*x*_^ is a convolutional deep neural network [27, 28], designed as a deep autoencoder.

The generator *G*:  ℛ^*N*_*x*_^⟶ℛ^*N*_*x*_^ has the same convolutional structure with the discriminator's decoder except the initial inputs and the first fully connected layer. This causes the simplicity of architecture, and the training procedure becomes simpler. The input of the generator is the concatenation of **z** and **c**, where **z** ∈ [−1,1]^*N*_*z*_^ is sampled uniformly.

As a result, we construct the following loss functions from the networks:

(7) $$\begin{array}{c} \left\{\begin{array}{c} {ℒ}_{Q}=E_{z∼p\left(z\right)c∼p\left(c\right),x∼G\left(z,c\right)}\left[\log\;Q\left(\left.c\right|x\right)\right],\\ {ℒ}_{\text{D}}=ℒ\left(x\right)-k_{i}ℒ\left(G\left(z,c\right)\right),\\ {ℒ}_{\text{G}}=ℒ\left(G\left(z,c\right)\right)-\lambda {ℒ}_{Q},\\ k_{i+1}=k_{i}+\lambda_{\text{k}}\left(\gamma ℒ\left(x\right)-ℒ\left(G\left(z,c\right)\right)\right), \end{array}\right. \end{array}$$

where ℒ(**x**) is an autoencoder loss as in Equation (3).

We trained an autoencoder network for the discriminator loss ℒ_D_, a generator network for the generator loss ℒ_G_, where ℒ_*Q*_ regularizes the generator not to ignore latent code **c**. In our objectives, the meaning of *γ* can be preserved as in the BEGAN model because we update *k*_*i*_ regardless of the regularizer ℒ_*Q*_. Therefore, we will measure the convergence of GANs as in [3].

## 4. Experiments

The goal of experiments is to evaluate if our proposed method can learn interpretable representations from image datasets while assuring the newly produced images to maintain high visual quality. Therefore, our experiment consists of two parts. First, we show the generated images while changing the learned latent code **c** and fixing the latent variable **z** and interpret the results. To evaluate the learned interpretable codes, we measure the accuracy of the generated samples. Second, we measure whether IBEGAN converges to the equilibrium by inspecting the change of loss and using convergence measure.

### 4.1. Data Description

In this paper, we used two real-world image datasets: CelebA and LSUN bedroom.

**Figure 3:**
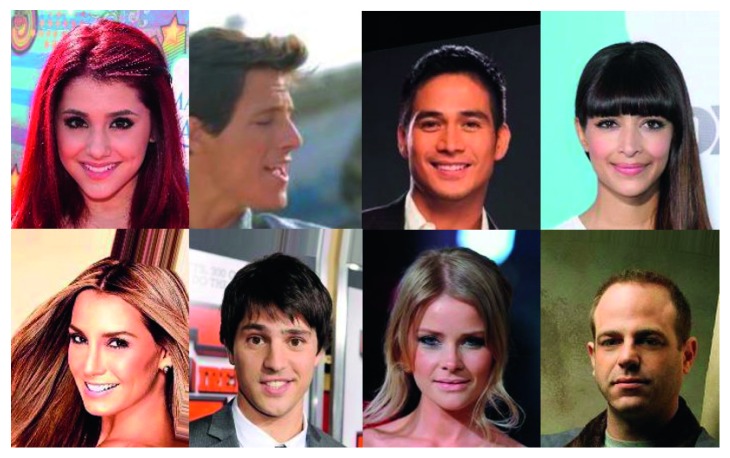
Samples of CelebA dataset.

Figure 3 shows CelebA dataset, which contains 202,599 images of celebrities. CelebA has a various facial poses, various races, and rotations around the camera axis [29]. Our proposed model generated the images that seem realistic and captured meaningful and manipulative representations.

**Figure 4:**
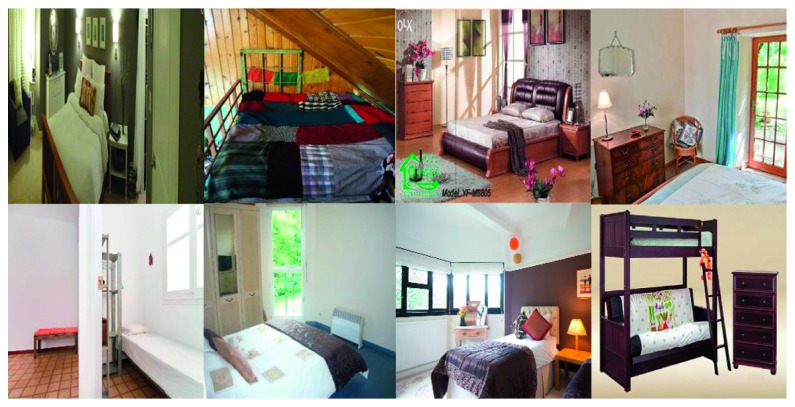
Samples of LSUN bedroom dataset.

Figure 4 shows LSUN bedrooms dataset, which contains about 3,033,042 images of bedrooms [30]. Though LSUN is a high resolution dataset with a lot of samples, the proposed model can successfully learn the representations and generate high-quality samples.

**Figure 5:**
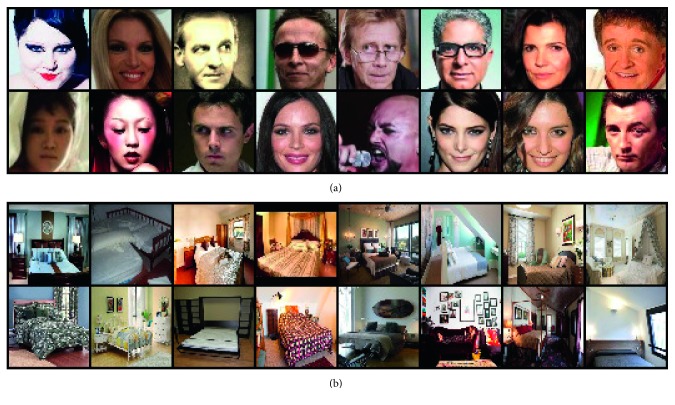
Resized face data and bedroom data that we used in this experiment.

In our experiments, we resized the image size of both CelebA data and LSUN bedroom data to 64 × 64, and with CelebA data, we used center crop of the image. The face data that were used in training are illustrated in Figure 5.

### 4.2. Experimental Design

In the experiments, we construct the network architectures of the discriminator and the generator as in Figures 1 and 2, where 3 × 3 convolutions with ELU (exponential linear units) layers are repeatedly applied at the previous outputs [31]. Each layer is repeated twice because the repetition of convolution can produce better visual quality. In encoder network, downsampling is done by subsampling with 2 strides. In decoder and generator networks, upsampling is done by using the nearest neighbor method. At the end of the encoder, the intermediate input is passed to a fully connected layer, and the embedding **h**, new input of the decoder is obtained. To capture the auxiliary distribution *Q*, the embedding **h** is passed to the next fully connected layer with the same output dimensions as the sum of the dimensions of the latent codes.

We estimated an auxiliary distribution *Q*(**c**|**x**) by using *Q* network which shares a lot of parts with the encoder network and constructed ℒ_*Q*_ from the softmax cross entropy between **c** and the estimated *Q*.

(8) $$\begin{array}{c} {ℒ}_{Q}=\frac{1}{M}\underset{i=1,...,N_{c}}{\sum }\underset{j=1,\ldots ,m_{i}}{\sum }\left(c_{i,j}\;*\;q_{i,j}\right), \end{array}$$

where *N*_**c**_ is the number of latent codes, *m*_*i*_ is the dimension of the latent code *i*, *M*=∑_*i*_*m*_*i*_, **c**_*i*,*j*_, is *j*‐th category of the latent code *i*, and **q**_*i*,*j*_=log *Q*(**c**_*i*_|**x**)_*j*_. This regularizer can make IBEGAN learn the meaningful latent codes. We will inspect this property in Section 4.3.1.

In [23], the effectiveness of disentangling factors was proved by measuring the accuracy, but IBEGAN cannot be verified in that way because the interpretable factors are learned in a unsupervised way. Although IBEGAN does not have any supervised label for the latent code unlike [23], the latent code should be determined to generate samples. Therefore, we used the latent code as the supervised label and estimated the most probable latent code from the auxiliary distribution. We generate artificial images $\tilde{x}$ by changing the latent codes $\tilde{c}$, estimating $Q\left(\left.c\right|\tilde{x}\right)$, and comparing the estimated codes $\hat{c}={\mathrm{argmax}}_{c}\;\;Q\left(\left.c\right|\tilde{x}\right)$ with the latent codes $\tilde{c}$. We calculate the accuracy of the estimation to provide the objective measure for the interpretability of our generated images.

To train the model, all two losses ℒ_D_ and ℒ_G_ in Equation (7) are minimized in an iteration, and the variable *k*_*i*_ ∈ [0,1] is updated to control how much to concentrate on the loss of the generator versus the loss of the data, **x**, during the gradient descent. We used the different basic settings of some hyperparameters for CelebA and LSUN bedroom datasets because images in CelebA datasets share more common features than LSUN. In both basic settings, we used a small batch size *m*=16 to avoid a memory error, and the Adam optimizer was used to minimize the losses [32]. In addition, diversity ratio *γ* was set to 0.5 and fixed. However, we set the dimension of hidden representation as 64 for CelebA and 128 for LSUN bedroom. In case of a learning rate of *kλ*_*k*_, we used 0.001 for CelebA dataset and 0.0005 for LSUN bedroom dataset.

We should carefully select a control parameter *λ* of the regularization term ℒ_*Q*_ in Equation (7). Unlike the InfoGAN model, we could not set *λ* to 1 because we used an autoencoder-based discriminator loss instead of a classifier-based loss. We initialized *λ* to 1/*M*^2^ and updated with the learning rates of the generator and the discriminator. However, unlike the learning rates, *λ* was increased when it was updated.

To measure the convergence of the model, we used a convergence measure that is proposed in [3].

(9) $$\begin{array}{c} {ℳ}_{\text{conv}}=ℒ\left(x\right)+\left|\gamma ℒ\left(x\right)-ℒ\left(G\left(z,c\right)\right)\right|. \end{array}$$

Because IBEGAN uses the proportion control algorithm, ℳ_conv_ is stabilized when the model reaches a final stage. If a model collapses, ℳ_conv_ cannot be stabilized. We analyze the convergence of our model from various loss values and this convergence measure in Section 4.3.2.

### 4.3. Experimental Results

#### 4.3.1. Interpretable Representation

In this section, we evaluate our generative model in terms of interpretable representation. From the learned latent codes, we can find meaningful features and tendencies in spite of the absence of any additional label information. In addition, we quantitatively verify that our model can generate the manipulated images by changing the interpretable latent code **c**.

**Figure 6:**
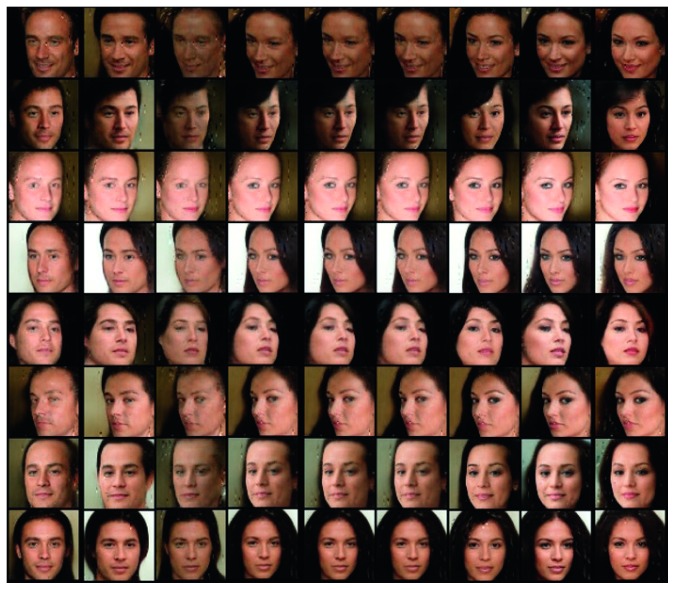
Varying **c**_1_ on CelebA dataset. We show the effect of the learned categorical latent factors on the outputs. This figure shows that a categorical code **c**_1_ can capture the gender of the face. We can see that the degree of masculinity of the image changes drastically as latent factor **c**_1_ changes.

**Figure 7:**
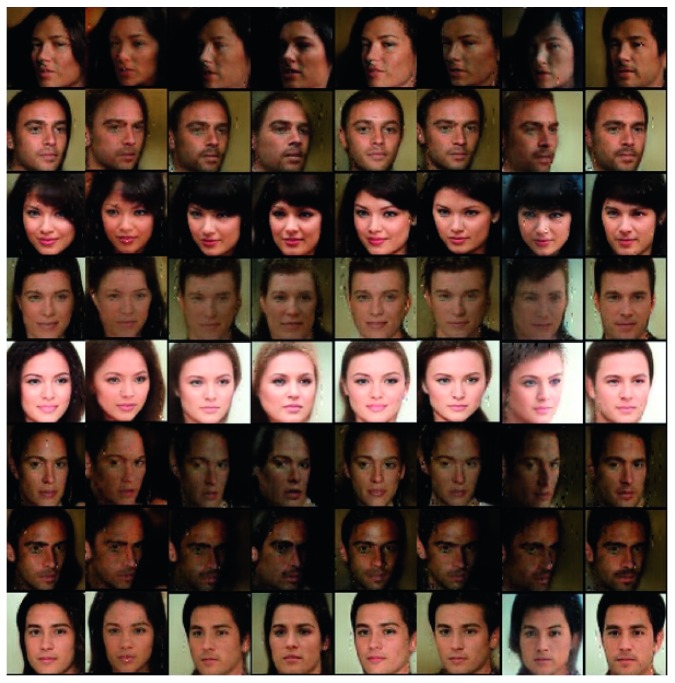
Varying **c**_2_ on CelebA dataset. This figure shows that a categorical code **c**_2_ can capture the hair style. As **c**_2_ changes, we can find that hair style changes while a person's face is same.

To generate the artificial face images like CelebA dataset, first we choose to model the latent codes with two categorical codes, **c**_1_ ~ Cat(*K*=9, *p*=1/9) and **c**_2_ ~ Cat(*K*=8, *p*=1/8). In this setting, our suggested model learns to represent latent factors as gender and hair style.  Figure 6 demonstrates that the variation of **c**_1_ is related to gender.  Figure 7 shows that the variation of **c**_2_ changes the hair style of the generated images. We can control the generated image of a person to look more manly by changing **c**_1_, and we can try different hair styles on images by controlling **c**_2_, which proves that our proposed model successfully learned interpretable representations.

Aforementioned experimental results showed the latent factors of hair style and masculinity. As a result, two factors are highly correlated because men and women usually have different hair styles. We also experimented with different level of categorical code in CelebA dataset. In this experiment, we selected latent codes with two categorical codes, **c**_1_ ~ Cat(*K*=10, *p*=1/10) and **c**_2_ ~ Cat(*K*=10, *p*=1/10). In this setting, both latent factors indicated independent feature of the image.

**Figure 8:**
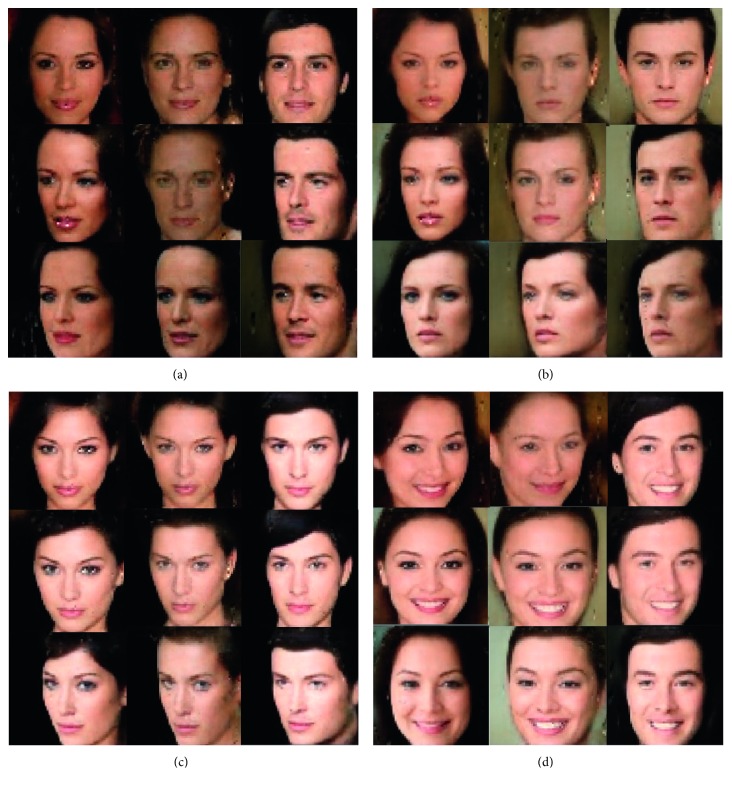
Variation of both **c**_1_ and **c**_2_ with same incompressible noise **z**. Adjoined nine images have same **z** with different latent codes **c**_1_ and **c**_2_. Row of the image denotes difference of **c**_1_ while column refers to **c**_2_.

Figure 8 shows the second experimental result in CelebA dataset. This image illustrates variation of both **c**_1_ and **c**_2_ with same incompressible noise **z**. Nine adjoined images are from same incompressible noise **z** and row denote varying **c**_1_ and column refers to **c**_2_.

We can find that **c**_1_ can capture the angle of the face, since generated faces tend to look at the left side as **c**_1_ changes. Also, **c**_2_ refers to masculinity since generated image's gender differs as **c**_2_ changes. Moreover, we found that image with same **z** has common in its background, skin color, and facial expressions such as smile. We can conclude that incompressible noise **z** denotes overall air of the image.

Two experiments in CelebA showed that interpretable representations in various latent codes **c**_1_, **c**_2_, …, **c**_*k*_, can be either independent or dependent because our model learns the latent codes without determining the desirable factors. In our first experiments in CelebA, two latent codes were correlated because the first code captured masculinity and the second code denoted hair style. In the second experiment settings, two latent codes captured hair style and angle of the face. Two latent codes in the second experiments were independent.

**Figure 9:**
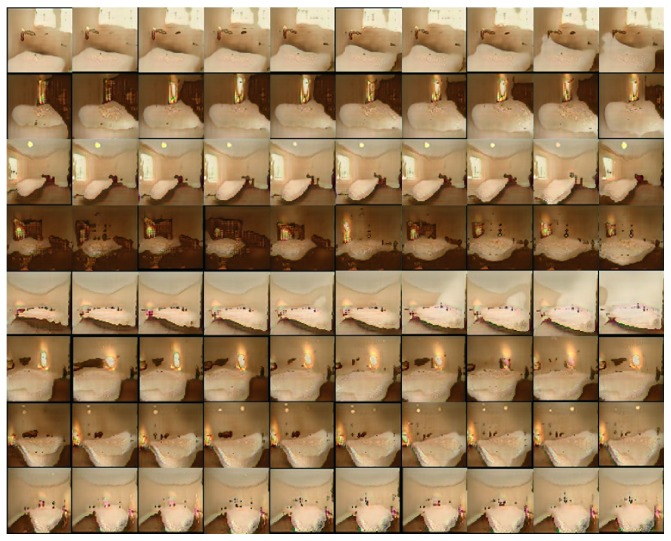
Varying *c* on LSUN dataset. The size of bed changes drastically.

We also implemented our suggested model to LSUN bedroom dataset, and the result is illustrated in Figure 9. In this experiment, we used latent codes with single categorical code with ten dimensions, **c**_1_ ~ Cat(*K*=10, *p*=1/10). We generated the images by fixing 10 different incompressible noises **z** and changing the one-hot encoding of the latent code **c**_1_. Therefore, in Figure 9, rows refer the same **z** and columns refer the same **c**_1_. In this experiment, **c**_1_ captures size of bed in each image. We can see in Figure 9 that size of bed changes as latent code **c**_1_ changes. In addition, similarly to CelebA, the incompressible noise **z** can capture overall air of the image.

In this section, we also verify the effectiveness of the interpretable latent codes. We generate the new images and calculate the auxiliary distribution *Q*(**c**|**x**). To generate an image using generator network, feeding the latent codes is required. Therefore, we can naturally obtain the label information of the generated image.  Table 1 illustrates the classification results based on auxiliary distribution *Q*(**c**|**x**). We repeated the experiments 100 times and provided the average and the standard deviation of the accuracy.

In Table 1, the first and the second rows illustrate the accuracy of the individual factor whereas the last row illustrates the accuracy of two factors at the same time. As expected, our learned interpretable codes are well reflected in the generated images and showed high accuracies and small standard deviations in all cases.

#### 4.3.2. Convergence of Models

In this section, we demonstrate that the IBEGAN model can produce high visual quality and be stably trained. These properties come from the advantage of the BEGAN model.

**Figure 10:**
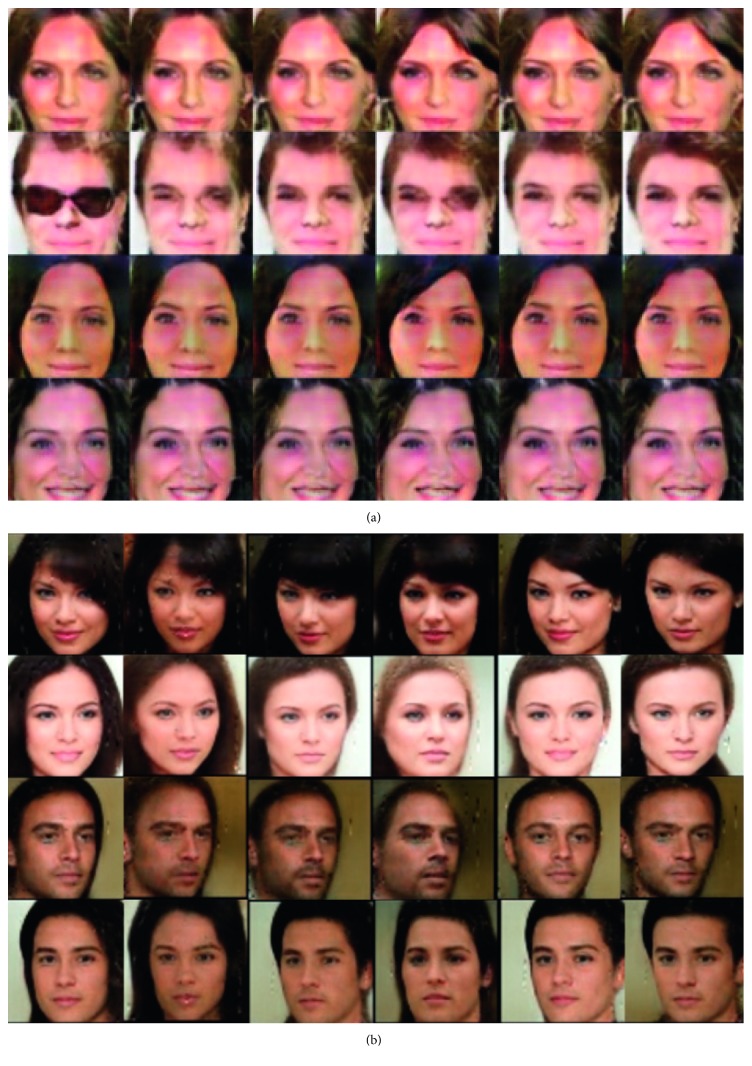
Comparison of our suggested model IBEGAN with previous model InfoGAN. Both models captured variation of hair style by changing latent code **c**_1_. (a) Generated images in InfoGAN. (b) Generated images in IBEGAN (Suggested model).

We aim not only to learn interpretable representations but also to obtain high-quality images. To verify this, we compared generated images of CelebA dataset from ours with images from the InfoGAN model. This result is illustrated in Figure 10. Both generated images changes hair styles by changing latent code **c**_1_. We can see that our suggested model shows better image quality compared to the previous model.

**Figure 11:**
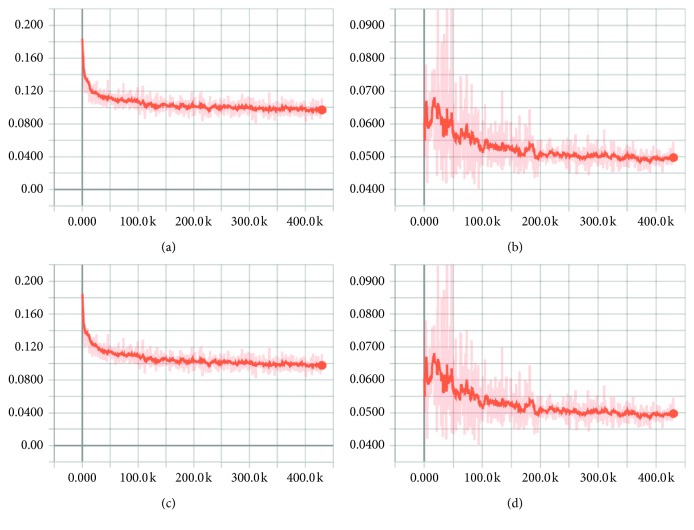
Various losses. (a) Discriminator loss. (b) Generator loss. (c) Autoencoder loss for real data. (d) Autoencoder loss for fake data.

To inspect the convergence of our model, we illustrate all loss values in Figure 11. We constructed the generator and discriminator losses based on autoencoder losses of real and fake data. Therefore, we drew the discriminator loss in Figure 11(a), the generator loss in Figure 11(b), the autoencoder loss of real data in Figure 11(c), and the autoencoder loss of fake data in Figure 11(d).

From the plots, we found that the pattern of the discriminator loss is similar to the autoencoder loss of real data and the pattern of the generator loss is similar to the autoencoder loss of fake data. It is because we set small *λ*, *k*_0_, and *λ*_*k*_. While the discriminator loss was stably declining, the generator loss was unstable in the beginning of the training but eventually stabilized. In this experiment, we set the balance parameter *γ* to 0.5. When the model converges, *γ* satisfies the balancing equation (4), where *γ* is the ratio of ℒ(*G*(**z**, **c**)) to ℒ(**x**). At the end of the iteration, the autoencoder loss of real data is about twice the autoencoder loss of fake data.

**Figure 12:**
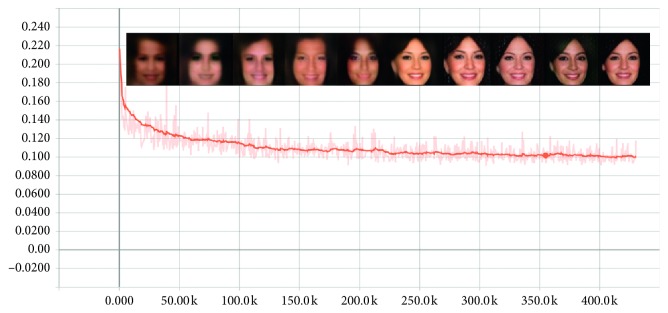
The convergence measure ℳ_conv_.

In addition, we evaluate the convergence of our model with the convergence measure (9).  Figure 12 shows the change of generated images with ℳ_conv_. The fidelity of image is low when ℳ_conv_ is high. Also, when ℳ_conv_ become stabilized, the images hardly change. We find that the IBEGAN model stably converges.

## 5. Conclusion

This paper proposed a meta-algorithm called IBEGAN. IBEGAN does not require any kind of supervision and is still able to learn interpretable representations. In addition, the IBEGAN model can have a simple training procedure and generate high-quality images by introducing equilibrium concept and proportional control theory as in the BEGAN model. In the experiment, we can obtain produced images that maintain the high visual quality, and the images can be manipulated as desired by changing the values of latent codes.

In this study, we used an autoencoder model to sophisticate the discriminator, but we should additionally construct the generator network unlike the BEGAN model because the deterministic autoencoder model cannot control the distribution of hidden representation. Therefore, the IBEGAN model can be extended to sharing generator network with the encoder network by using variational autoencoder.

However, the IBEGAN model had a difficulty in learning latent codes independently. This model can be extended to adding a regularization term that could make latent factors independently. In this way, IBEGAN model would produce more robust disentangling latent codes that are independent.

## Figures and Tables

**Table 1:** The accuracy of the estimation of the interpretable codes based on *Q*(**c**|**x**).

Latent factor	Accuracy	Standard deviation
**c**_1_	0.9865	0.0051
**c**_2_	0.9930	0.0048
**c**_1_ and **c**_2_	0.9796	0.0080

## Data Availability

The data used to support the findings of this study are available from the corresponding author upon request.
